# Supplementary material for: Unveiling the Bioactive Potential of the Invasive Jellyfish Phyllorhiza punctata Through Integrative Transcriptomic and Proteomic Analyses
Source: Biomolecules. 2025 Aug 4;15(8):1121. doi: 10.3390/biom15081121 (PMC12383608; doi:10.3390/biom15081121)
Supplement: Supplementary file 1 [file biomolecules-15-01121-s001.zip › Suplementary Material/FileS3/PEPFOLD4/XP_065051196_924/clusters.html]

 PEP-FOLD clusters


Download archive of all models  here   

|  |  |  |
| --- | --- | --- |
| # | file | sOPEP |
| # cluster 1 - Size: 2 |  |  |
|  | XP\_065051196\_924-model1 | -7.18859 |
|  | XP\_065051196\_924-model1.1 | -6.33145 |
| # cluster 2 - Size: 2 |  |  |
|  | XP\_065051196\_924-model2 | -7.08646 |
|  | XP\_065051196\_924-model2.1 | -5.9153 |
| # cluster 3 - Size: 2 |  |  |
|  | XP\_065051196\_924-model3 | -6.98831 |
|  | XP\_065051196\_924-model3.1 | -5.50155 |
| # cluster 4 - Size: 1 |  |  |
|  | XP\_065051196\_924-model4 | -6.93317 |
| # cluster 5 - Size: 2 |  |  |
|  | XP\_065051196\_924-model5 | -6.92729 |
|  | XP\_065051196\_924-model5.1 | -6.08211 |
| # cluster 6 - Size: 1 |  |  |
|  | XP\_065051196\_924-model6 | -6.86955 |
| # cluster 7 - Size: 1 |  |  |
|  | XP\_065051196\_924-model7 | -6.84992 |
| # cluster 8 - Size: 2 |  |  |
|  | XP\_065051196\_924-model8 | -6.64152 |
|  | XP\_065051196\_924-model8.1 | -6.2783 |
| # cluster 9 - Size: 5 |  |  |
|  | XP\_065051196\_924-model9 | -6.44474 |
|  | XP\_065051196\_924-model9.1 | -6.18399 |
|  | XP\_065051196\_924-model9.2 | -5.29222 |
|  | XP\_065051196\_924-model9.3 | -5.14636 |
|  | XP\_065051196\_924-model9.4 | -4.96254 |
| # cluster 10 - Size: 1 |  |  |
|  | XP\_065051196\_924-model10 | -6.25363 |
| # cluster 11 - Size: 1 |  |  |
|  | XP\_065051196\_924-model11 | -6.21314 |
| # cluster 12 - Size: 1 |  |  |
|  | XP\_065051196\_924-model12 | -6.17695 |
| # cluster 13 - Size: 1 |  |  |
|  | XP\_065051196\_924-model13 | -6.00141 |
| # cluster 14 - Size: 1 |  |  |
|  | XP\_065051196\_924-model14 | -5.94458 |
| # cluster 15 - Size: 1 |  |  |
|  | XP\_065051196\_924-model15 | -5.85436 |
| # cluster 16 - Size: 4 |  |  |
|  | XP\_065051196\_924-model16 | -5.78736 |
|  | XP\_065051196\_924-model16.1 | -5.57227 |
|  | XP\_065051196\_924-model16.2 | -5.42165 |
|  | XP\_065051196\_924-model16.3 | -5.35962 |
| # cluster 17 - Size: 3 |  |  |
|  | XP\_065051196\_924-model17 | -5.74226 |
|  | XP\_065051196\_924-model17.1 | -5.04865 |
|  | XP\_065051196\_924-model17.2 | -4.88494 |
| # cluster 18 - Size: 1 |  |  |
|  | XP\_065051196\_924-model18 | -5.69834 |
| # cluster 19 - Size: 2 |  |  |
|  | XP\_065051196\_924-model19 | -5.61831 |
|  | XP\_065051196\_924-model19.1 | -5.30724 |
| # cluster 20 - Size: 9 |  |  |
|  | XP\_065051196\_924-model20 | -5.57502 |
|  | XP\_065051196\_924-model20.1 | -5.30607 |
|  | XP\_065051196\_924-model20.2 | -5.20997 |
|  | XP\_065051196\_924-model20.3 | -5.09513 |
|  | XP\_065051196\_924-model20.4 | -5.08166 |
|  | XP\_065051196\_924-model20.5 | -5.08044 |
|  | XP\_065051196\_924-model20.6 | -5.03307 |
|  | XP\_065051196\_924-model20.7 | -5.01965 |
|  | XP\_065051196\_924-model20.8 | -4.88898 |
| # cluster 21 - Size: 1 |  |  |
|  | XP\_065051196\_924-model21 | -5.56902 |
| # cluster 22 - Size: 5 |  |  |
|  | XP\_065051196\_924-model22 | -5.42424 |
|  | XP\_065051196\_924-model22.1 | -5.30058 |
|  | XP\_065051196\_924-model22.2 | -5.12735 |
|  | XP\_065051196\_924-model22.3 | -4.97638 |
|  | XP\_065051196\_924-model22.4 | -4.94288 |
| # cluster 23 - Size: 1 |  |  |
|  | XP\_065051196\_924-model23 | -5.3918 |
| # cluster 24 - Size: 4 |  |  |
|  | XP\_065051196\_924-model24 | -5.34254 |
|  | XP\_065051196\_924-model24.1 | -5.33854 |
|  | XP\_065051196\_924-model24.2 | -5.3115 |
|  | XP\_065051196\_924-model24.3 | -5.18241 |
| # cluster 25 - Size: 1 |  |  |
|  | XP\_065051196\_924-model25 | -5.3348 |
| # cluster 26 - Size: 4 |  |  |
|  | XP\_065051196\_924-model26 | -5.30088 |
|  | XP\_065051196\_924-model26.1 | -5.28836 |
|  | XP\_065051196\_924-model26.2 | -5.27551 |
|  | XP\_065051196\_924-model26.3 | -5.12572 |
| # cluster 27 - Size: 1 |  |  |
|  | XP\_065051196\_924-model27 | -5.28136 |
| # cluster 28 - Size: 1 |  |  |
|  | XP\_065051196\_924-model28 | -5.26998 |
| # cluster 29 - Size: 5 |  |  |
|  | XP\_065051196\_924-model29 | -5.26557 |
|  | XP\_065051196\_924-model29.1 | -5.10637 |
|  | XP\_065051196\_924-model29.2 | -5.0816 |
|  | XP\_065051196\_924-model29.3 | -5.03465 |
|  | XP\_065051196\_924-model29.4 | -4.8876 |
| # cluster 30 - Size: 5 |  |  |
|  | XP\_065051196\_924-model30 | -5.25806 |
|  | XP\_065051196\_924-model30.1 | -5.19016 |
|  | XP\_065051196\_924-model30.2 | -5.14982 |
|  | XP\_065051196\_924-model30.3 | -5.00399 |
|  | XP\_065051196\_924-model30.4 | -4.92294 |
| # cluster 31 - Size: 2 |  |  |
|  | XP\_065051196\_924-model31 | -5.25006 |
|  | XP\_065051196\_924-model31.1 | -5.09032 |
| # cluster 32 - Size: 1 |  |  |
|  | XP\_065051196\_924-model32 | -5.23817 |
| # cluster 33 - Size: 1 |  |  |
|  | XP\_065051196\_924-model33 | -5.21265 |
| # cluster 34 - Size: 2 |  |  |
|  | XP\_065051196\_924-model34 | -5.20644 |
|  | XP\_065051196\_924-model34.1 | -5.19697 |
| # cluster 35 - Size: 3 |  |  |
|  | XP\_065051196\_924-model35 | -5.20113 |
|  | XP\_065051196\_924-model35.1 | -5.19147 |
|  | XP\_065051196\_924-model35.2 | -5.03634 |
| # cluster 36 - Size: 1 |  |  |
|  | XP\_065051196\_924-model36 | -5.19473 |
| # cluster 37 - Size: 3 |  |  |
|  | XP\_065051196\_924-model37 | -5.18722 |
|  | XP\_065051196\_924-model37.1 | -5.14227 |
|  | XP\_065051196\_924-model37.2 | -5.04306 |
| # cluster 38 - Size: 1 |  |  |
|  | XP\_065051196\_924-model38 | -5.11207 |
| # cluster 39 - Size: 3 |  |  |
|  | XP\_065051196\_924-model39 | -5.09605 |
|  | XP\_065051196\_924-model39.1 | -5.08914 |
|  | XP\_065051196\_924-model39.2 | -4.7341 |
| # cluster 40 - Size: 1 |  |  |
|  | XP\_065051196\_924-model40 | -5.0679 |
| # cluster 41 - Size: 3 |  |  |
|  | XP\_065051196\_924-model41 | -5.06758 |
|  | XP\_065051196\_924-model41.1 | -5.00129 |
|  | XP\_065051196\_924-model41.2 | -4.9056 |
| # cluster 42 - Size: 1 |  |  |
|  | XP\_065051196\_924-model42 | -5.06638 |
| # cluster 43 - Size: 1 |  |  |
|  | XP\_065051196\_924-model43 | -5.05233 |
| # cluster 44 - Size: 1 |  |  |
|  | XP\_065051196\_924-model44 | -5.02603 |
| # cluster 45 - Size: 1 |  |  |
|  | XP\_065051196\_924-model45 | -5.01903 |
| # cluster 46 - Size: 2 |  |  |
|  | XP\_065051196\_924-model46 | -4.97468 |
|  | XP\_065051196\_924-model46.1 | -4.88289 |
| # cluster 47 - Size: 1 |  |  |
|  | XP\_065051196\_924-model47 | -4.91539 |
| # cluster 48 - Size: 1 |  |  |
|  | XP\_065051196\_924-model48 | -4.9116 |
|  |  |  |
